# Biotechnological production of crocetin and crocins using a carotenoid cleavage dioxygenase (CCD4) from *Nyctanthes arbor-tristis*

**DOI:** 10.3389/fpls.2025.1671592

**Published:** 2025-10-17

**Authors:** Lucía Morote, Elena Moreno Giménez, Alberto José López Jiménez, Ángela Rubio-Moraga, Verónica Aragonés, Oussama Ahrazem, José-Antonio Daròs, Lourdes Gómez-Gómez

**Affiliations:** ^1^ Instituto Botánico, Departamento de Ciencia y Tecnología Agroforestal y Genética, Universidad de Castilla-La Mancha, Albacete, Spain; ^2^ Escuela Técnica Superior de Ingeniería Agronómica y de Montes y Biotecnología, Departamento de Ciencia y Tecnología Agroforestal y Genética, Universidad de Castilla-La Mancha, Albacete, Spain; ^3^ Instituto de Biología Molecular y Celular de Plantas (Consejo Superior de Investigaciones Científicas-Universitat Politècnica de València), Valencia, Spain; ^4^ Facultad de Farmacia, Universidad de Castilla-La Mancha, Albacete, Spain

**Keywords:** aqueous two-phase, crocetin, carotenoid cleavage dioxygenase, *Buddleja*, *Crocus*, *Verbascum*, metabolic engineering

## Abstract

Crocins are hydrophilic crocetin esters composed of a linear chain with glucose molecules added at the end. Crocins and crocetin are used as cosmetic agents and effective pharmaceuticals for the treatment of several diseases. Crocetin dialdehyde, an immediate precursor of crocetin, is derived by C7-C8(C7'-C8′) cleavage of carotenoids, which is synthesized in a few plant species including *Crocus sativus* or *Nyctanthes arbor-tristis*. We investigated the genome of *N. arbor-tristis* to identify the enzyme responsible for the biosynthesis of crocetin dialdehyde in this plant and showed that an enzyme from the CCD4 subfamily catalyzed the cleavage of zeaxanthin to produce this apocarotenoid. This enzyme, NatCCD4.1, was further used for the microbial production of crocetin dialdehyde in a two-phase culture system resulting in a titer of 109.2 ± 3.23 mg/L, which is the highest crocetin dialdehyde yield reported in bacteria so far, higher than that obtained when using the enzyme from *C. sativus*, CsCCD2. In addition, a viral vector derived from tobacco etch virus was used to express NatCCD4.1 in *Nicotiana benthamiana* plants, triggering a crocin accumulation of 2.32 ± 0.69 mg/g dry weight, with 96.61% reduction in zeaxanthin levels, together with a decrease in chlorophylls resulting in a bright yellow pigmentation of infected leaves. Our results offer new insights into the biosynthesis of crocins in crocin-producing species. Additionally, NatCCD4.1 proves to be an excellent tool for metabolic engineering, enhancing crocetin and crocin production in various heterologous systems.

## Introduction

**Figure 1:**
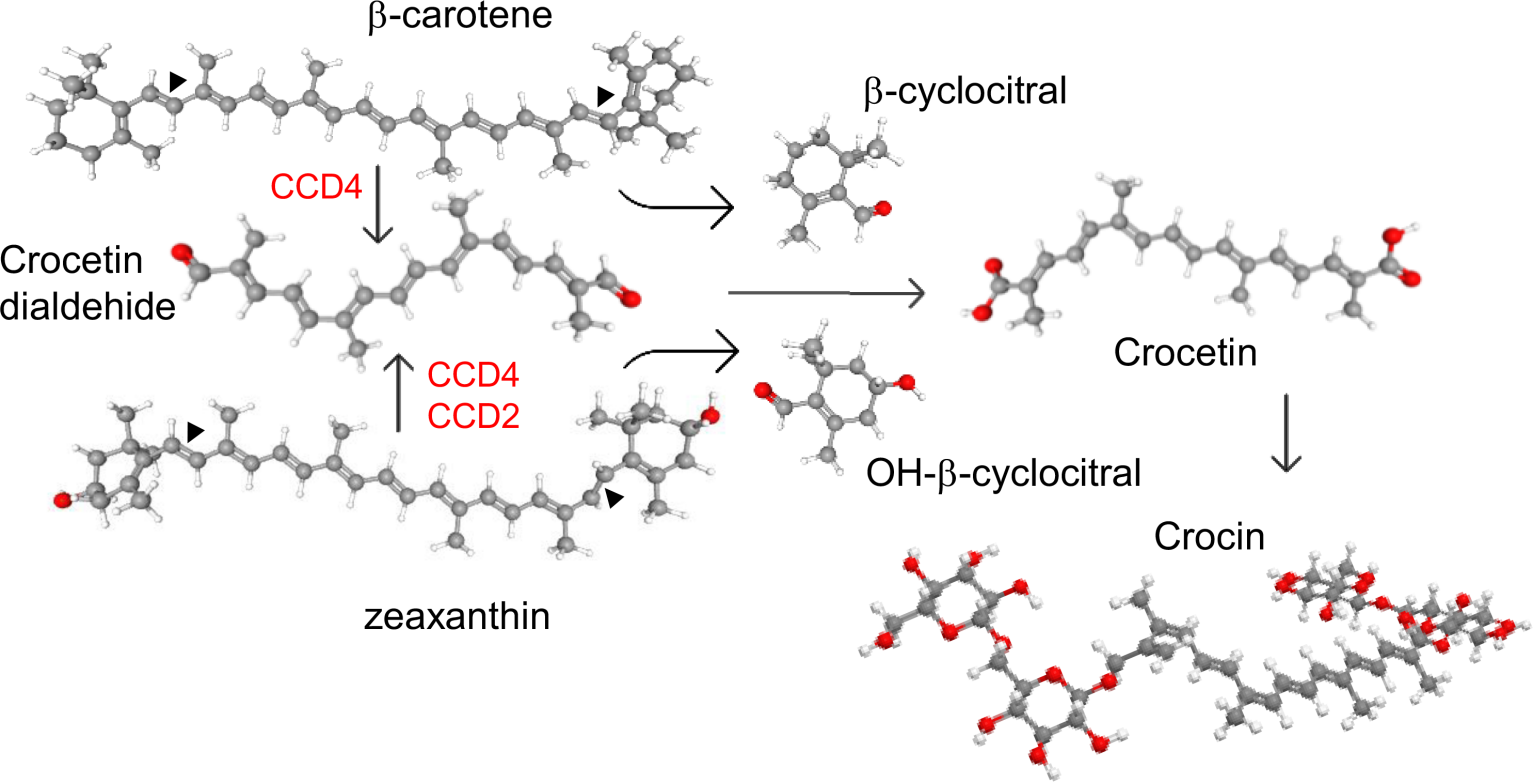
Cleavage activities of CCD enzymes on β-carotene and zeaxanthin, producing different apocarotenoid products. In red are shown the Carotenoid Cleavage dioxygenase enzymes (CCD) subfamilies involved in the C7-C8(C7'-C8') cleavage off carotenoids.

Crocetin is a class of lipophilic isoprenoid molecule composed of a polyunsaturated chain, with two alcohol (crocetindiol), two aldehyde (crocetindial), two carboxylic acid (crocetin), or esters (crocins) functional groups (**Figure 1**) (Song et al., 2021). Crocetin and crocins (Cerdá-Bernad et al., 2022) display a broad range of therapeutic properties due to their antioxidant and anti-inflammatory activities and are also known to reduce the risk of certain cancers (Guo et al., 2021; Koch et al., 2024). Crocetin and crocins have been tested in clinical trials on depression, anxiety, and other brain disorders as Alzheimer and Parkinson (Bian et al., 2020). These advantageous characteristics are fueling the expansion of crocetin esters in a range of pharmaceutical goods (Ali et al., 2022). The crocetin and crocin market size has been estimated to be about 4.7 billion USD worldwide and is expected to grow at a compound annual growth rate of 5.16% from 2024 to 2030. The crocetin biosynthesis pathway is present only in plants, and *Crocus sativus* stigmas and *Gardenia jasminoides* fruits are the principal commercial source of these compounds. Other plants are known to produce crocetin and crocins, but at lower levels, as *Buddleja davidii* (Ahrazem et al., 2017), *Verbascum* sp. (Morote et al., 2024 and *Nyctanthes arbor-tristis* (Gadgoli and Shelke, 2010). The actual production of crocetin and corresponding esters cannot meet the market demand due to the high costs associated to cultivation and extraction from the main plant source (Ahrazem et al., 2015). In addition, prevailing environmental conditions, such as global warming and drought, have exacerbated the production of these valuable metabolites (Pirasteh-Anosheh et al., 2023). To meet the increasing demand for crocetin and crocins, several attempts in different heterologous systems using the enzymes from crocetin biosynthesis in saffron, *Buddleja* and gardenia have been reported with variable results (Gómez-Gómez et al., 2023; Zhou et al., 2023). New biotechnological processes for the improved production of crocetin and crocins using metabolically engineered microorganism and fast-growing plants with highly active enzymes are needed. For such purpose, discovery, and characterization of new enzymes for crocetin biosynthesis is a straightforward strategy.

The enzymes involved in the biosynthesis of crocetin belong to the carotenoid cleavage dioxygenase (CCD) family. These enzymes catalyze the cleavage of carotenoids within the plastids, producing apocarotenoid compounds that, after oxidation and glucosylation, accumulate in the vacuoles (Ahrazem et al., 2016a; Liu et al., 2020b). These enzymes vary in substrate specificity and cleavage sites, generating a wide range of apocarotenoids (Zheng et al., 2021). The CCD enzymes involved in crocetin biosynthesis cleave carotenoids at the C7–C8 (C7′–C8′) double bonds. The enzyme CsCCD2L from *C. sativus* produces crocetin dialdehyde by cleaving zeaxanthin (Frusciante et al., 2014; Ahrazem et al., 2016b). In species such as gardenia, *Buddleja*, and *Verbascum*, CCDs from the CCD4 subfamily catalyze crocetin dialdehyde biosynthesis and can recognize multiple carotenoid substrates, including lycopene, zeaxanthin, and β-carotene (Ahrazem et al., 2017; Xu et al., 2020; Morote et al., 2024) (**Figure 1**). Similarly, a CCD4 from *Bixa orellana*, which does not accumulate crocetin or crocins, has been shown to catalyze the C7–C8 cleavage of lycopene (Frusciante et al., 2022).

Crocins accumulate in the colored tubular calyx of the flower (Bhuskat et al., 2007; Gadgoli and Shelke, 2010) that also produces safranal (Siriwardena and Arambewela, 2014). The total concentration of crocins in the calix reaches 35.57% (w/w) and have been used as a potential wound healing phytoconstituent (Varadkar and Gadgoli, 2022), which also shows hypoglycemic and hypolipidemic properties (Rangika et al., 2015). Recently, genome and transcriptome analyses of *N. arbor-tristis* have identified 15 contigs homologous to CCDs that could be involved in crocetin biosynthesis in this species (Patil et al., 2023). In this study, we have identified a novel CCD4 enzyme from *N. arbor-tristis* involved in the biosynthesis of crocetin. Functional analysis of this enzyme revealed its C7–C8 (C7′–C8′) cleavage activity on zeaxanthin. To advance the biotechnological exploitation of this enzyme, we employed a two-phase culture system using dodecane for the production of crocetin dialdehyde in *Escherichia coli* and a virus-driven system to produce crocins in *Nicotiana benthamiana*.

## Material and methods

### Identification of CCD4 genes by bioinformatic analyses

A transcriptome from flowers of *N. arbor-tristis* and the genome sequence (Patil et al., 2023) were searched for contigs bearing homology to genes encoding for enzymes of the CCD4 subfamily using BLAST (https://blast.ncbi.nlm.nih.gov/Blast.cgi). Phylogenetic trees were generated using MEGA version 11.0.10 with the maximum-likelihood method (https://megasoftware.net/) and bootstrap tests replicated 5000 times. Prediction of subcellular localization was obtained using the DeepLoc-1.0 software (https://services.healthtech.dtu.dk/services/DeepLoc-1.0/). The 3D structures were predicted using the Phyre2 software at intensive mode (http://www.sbg.bio.ic.ac.uk/phyre2/), and Chimera X (https://www.cgl.ucsf.edu/chimerax/).

### Gene synthesis and cloning

DNA sequences were synthesized by the gene synthesis service of NZYtec (https://www.nzytech.com/), and used as templates for amplification with specific primers (**Supplementary Table S1**) for cloning in the expression vector pTHIO-Dan1 using In-Fusion assembly (Takara Bio, Europe), and for cloning in a viral vector derived from tobacco etch virus (TEV) (**Supplementary Table S1**) by Gibson DNA assembly (New England Biolabs), as previously described (Morote et al., 2024). The obtained plasmids, pTHIO-NatCCD4.1, pTHIO-NatCCD4.2 and pGTEV-NatCCD4.1, were sequenced using an automated DNA sequencer (ABI PRISM 3730xl, Perkin Elmer, Macrogen Inc., Seoul, Korea).

### Analysis of enzymatic activity in *E. coli*

*E. coli* strain BL21 (DE3) was the host strain used for the activity assays using different substrates. Competent cells were transformed with the plasmids PAC-LYC, PAC-ZEAX, and PAC-BETA (https://www.addgene.org) to produce lycopene, zeaxanthin, and β-carotene. The positive transformants were further used for the introduction of pTHIO-NatCCD4.1, pTHIO-NatCCD4.2, pTHIO-CsCCD2 (Frusciante et al., 2014) and the empty vector pTHIO-Dan1. Double transformants were cultured overnight at 37°C in 3 mL LB medium supplemented with ampicillin (100 µg/mL) and chloramphenicol (60 µg/mL). Cultures for activity assays were carried out in 2x YT (16 g of tryptone, 10 g of yeast extract, 5 g of NaCl) or in Terrific Broth (24 g of yeast extract, 20 g of tryptone, and 4 mL of glycerol per liter, and 0.017 M KH_2_PO_4_, and 0.072 M K_2_HPO_4_) supplemented with ampicillin (50 µg/mL) and chloramphenicol (30 µg/mL) using a shaking incubator at 30°C and 200 revolution per min (rpm). Expression was induced with 2% (w/v) arabinose. For the two-phase culture for apocarotenoid production, n-dodecane was layered over the culture medium (Terrific Broth) immediately after arabinose induction using different final concentrations (9%, 16%, 23% and 28%) and incubation times (6, 24, 36 and 72 h) after arabinose addition.

### Analysis of carotenoids and apocarotenoids

Carotenoid and apocarotenoid products were extracted from bacterial cell pellets as previously described (Gomez-Gomez et al., 2020). In the two-phase culture system with a n-dodecane overlay, the upper organic phase containing the CCD cleavage products was collected and centrifuged for 10 min at 12,000 x g to remove cellular remains and directly injected for apocarotenoid analyses. The pigments extracted from the cell pellets and those present in the n-dodecane phases were analyzed by high-performance liquid chromatography with diode-array detection (HPLC-DAD)(Agilent technologies 1100 series) at detection wavelengths of 450 nm and using a YMC C30 (250 × 4.6 mm, 5µm) column (Waters, Milford, USA). The mobile phases were 98:2 methanol (A), 95:5 methanol (B) and 100% methyl *tert*-butyl ether. The column was developed at a flow rate of 1 mL min^−1^ with the following gradient elution: 80% A, 20% C at 0 min, followed by linear gradient to 60% A, 40% C to 3 min at 4 min with gradient changing to 60% B, 40% C followed by a linear gradient to 0% B, 100% C by 12 min and return to initial conditions by 13 min. A re-equilibration (10 min) was carried out at initial conditions of 80% A, 20% C. A flow rate of 1 mL/min and column temperature of 40 °C were used. Crocetin dialdehyde (Cat. No. 18804, Sigma) was used as standard compound. The results are presented as means ± standard deviation (SD) from three independent experiments.

### Activity assays in *N. benthamiana*

The viral recombinant clone TEV-NatCCD4.1 consists of a wild-type TEV cDNA (GenBank DQ986288, containing the silent and neutral mutations G273A and A1119G), in which the NatCCD4.1 cDNA was inserted as the amino terminal cistron and was followed with an artificial NIaPro cleavage site to release the enzyme from the viral polyprotein. This viral clone was flanked by the 35S promoter and terminator sequences from the cauliflower mosaic virus (CaMV), in a binary vector derived from pCLEAN-G181 (Martí et al., 2020). The control construct TEV-GFP was previously described (Bedoya et al., 2012).

*Agrobacterium tumefaciens* C58C1 competent cells, containing the helper plasmid pCLEAN-S48, were transformed by electroporation with pGTEV-NatCCD4.1 or pGTEV-aGFP, as control. Transformants were selected in plates with 50 µg/mL kanamycin, 50 µg/mL rifampicin, and 7.5 µg/mL tetracycline. Selected clones were grown in liquid media and were prepared to infiltrate two leaves of one-month-old *N. benthamiana*. Inoculated plants were kept under controlled conditions in a growth chamber at 25°C under a 16/8 h day-night photoperiod. Leaf tissue was collected 14 days post-inoculation (dpi), frozen immediately, and lyophilized for further analysis for apocarotenoid and carotenoid content.

### Extraction and analysis of apocarotenoids and carotenoids from *N. benthamiana* leaves by TLC and HPLC-DAD

Lyophilized tissues were ground with a mixer mill MM400 (Retsch GmbH, Haan, Germany) in 2 mL tubes and 0.05 g used for polar and apolar extractions. For extraction of crocins, 50% methanol was added to the homogenate, mixed, vortexed, sonicated during 10 min in a water bath, and centrifuged 10 min at 10.000 x g. The supernatant was retained for crocins analyses by HPLC-DAD as previously described (Morote et al., 2024). The pellet was extracted with 2:1 methanol:chloroform, mixed for an additional 10 min and incubated in an ultrasound bath for an additional 10 min followed by centrifugation. The apolar phase containing carotenoids and chlorophylls was evaporated under N_2_ gas and the dried pellet was stored with the polar extracts at -80 °C until analysis by HPLC-DAD. All assays were performed in triplicate. The HPLC-DAD method used for the analysis and detection of carotenoids was the same described in the bacteria assay section. Metabolite identification was done by comparison of retention times, and UV-visible spectra using zeaxanthin, β-carotene, lycopene and lutein standards purchased from CaroteNature (Lupsingen, Switzerland).

Thin layer chromatography (TLC) for carotenoid and chlorophyll separation was performed with silica gel 60 F254 plates using petroleum ether:diethyl ether:acetone (30:15:15) as the mobile phase.

## Results

### Identification of a CCD4 enzyme in *N. arbor-tristis* involved in the synthesis of crocetin

**Figure 2:**
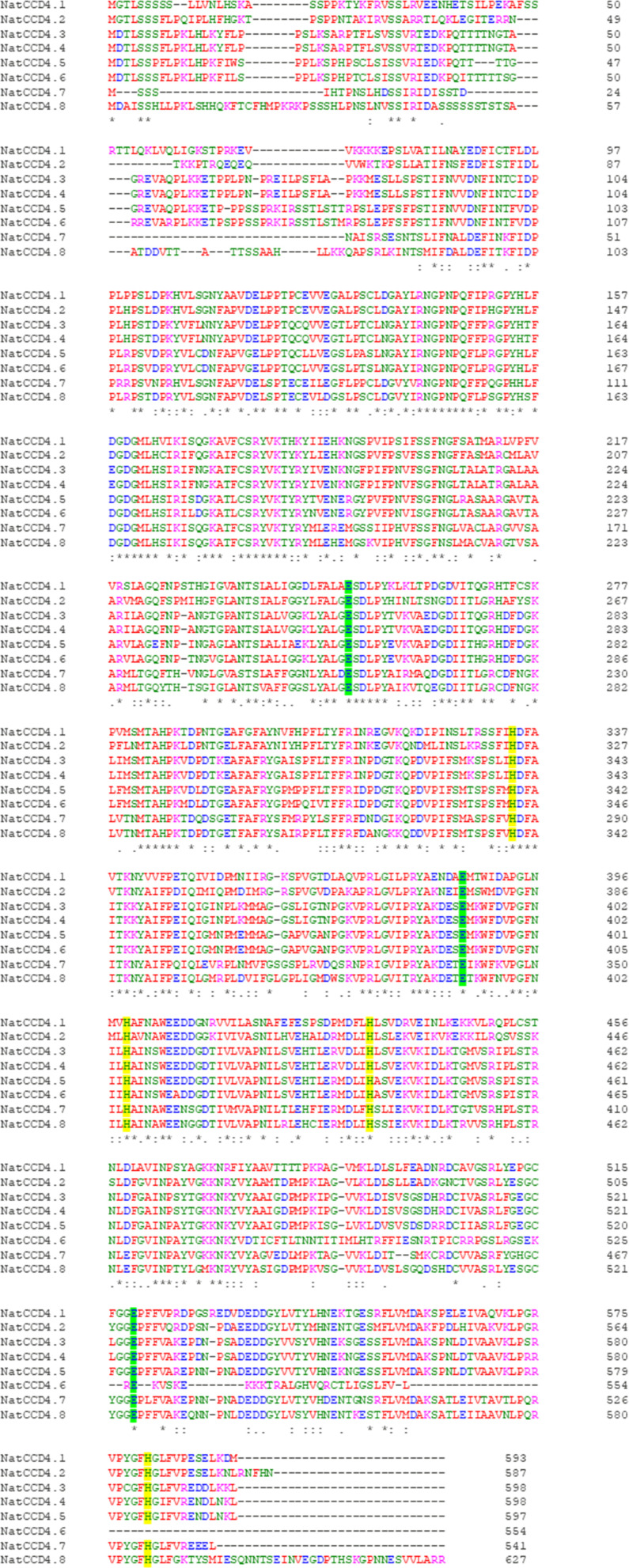
Amino acid sequence alignment of NatCCD4 enzymes. Conserved amino acid residues are depicted with asterisk. The amino acid residues involved in iron coordination are highlighted in yellow and green.

In a previously published transcriptome of *N. arbor-tristis* tissues (Patil et al., 2023), 15 contigs with a high identity to CCD4-encoding genes and expressed at high levels in flower tissue were identified. Of these 15 contigs, 11 of them were pedicel-specific with variable expression levels. Only two contigs out of the 15 encoded full-length CCD4 proteins. In order to get the complete sequences for all the other contigs, we analyzed the sequenced genome of *N. arbor-tristis* (Bioproject PRJEB46894). A total of 8 genes encoding CCD4 were identified and named from NatCCD4.1 to NatCCD4.8, which showed identities from 46.46% to 98.66% (**Figure 2** and **Supplementary Figure S1**). The identified contigs in the flower transcriptome correspond to genes *NatCCD4.1*, *3*, *4*, *5* and *6*, while no contigs correspond to genes *NatCCD4.2*, *7* and *8*. All the amnio acid sequences were analyzed for predicted localization (**Supplementary Figure S2**), and all proteins were predicted to be targeted to the plastid.

**Figure 3:**
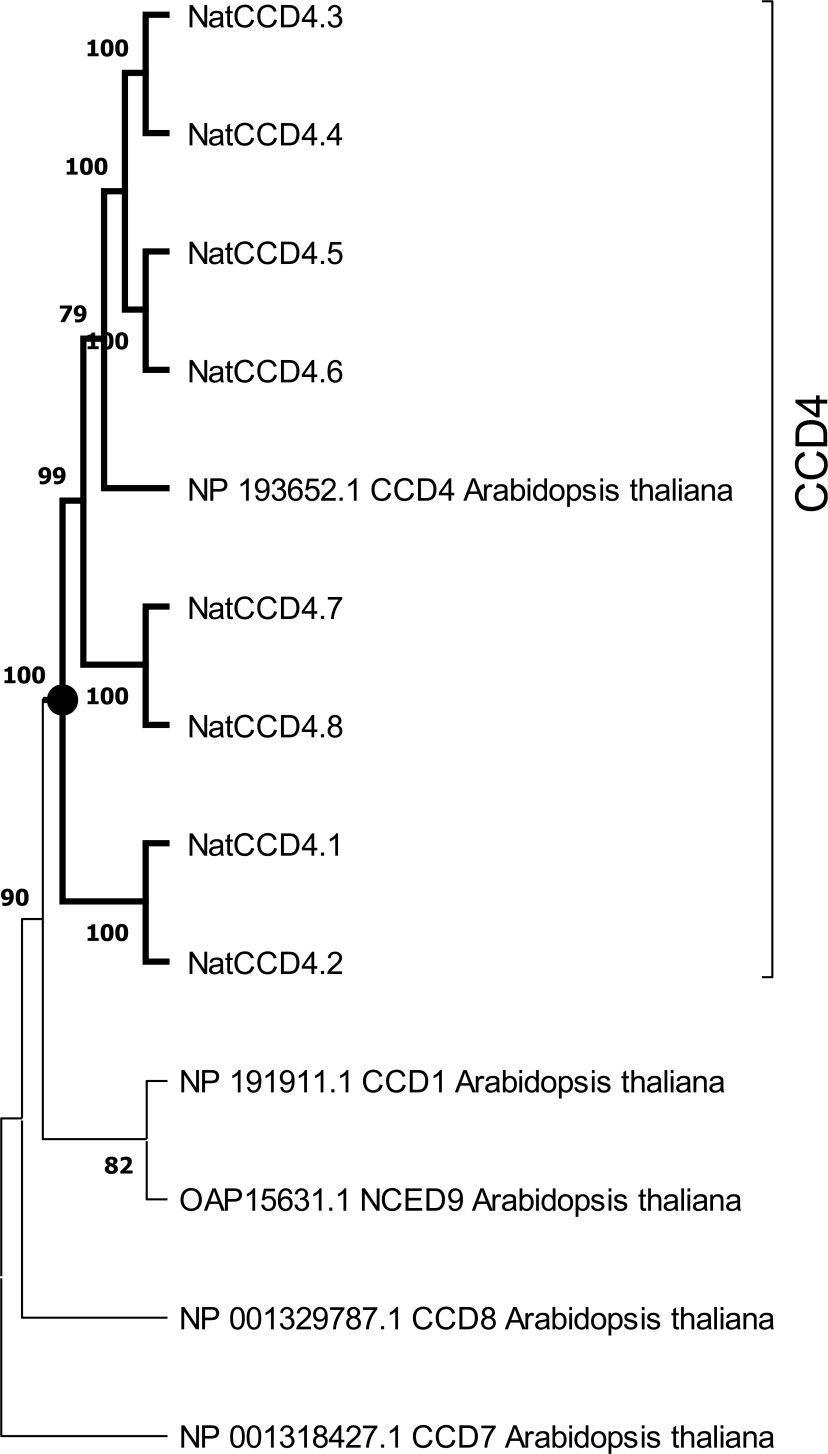
Phylogenetic tree of CCD4 amino acid sequences encoded by genes identified in the genome of *Nyctanthes arbor-tristis*, and CCDs and NCEDs of *Arabidopsis thaliana*. The cluster containing all the CCD4 enzymes is highlight in bold. Phylogenetic analysis was done using MEGA version 11.0.10 with the maximum-likelihood method (https://megasoftware.net/) and bootstrap tests replicated 5000 times.

**Figure 4:**
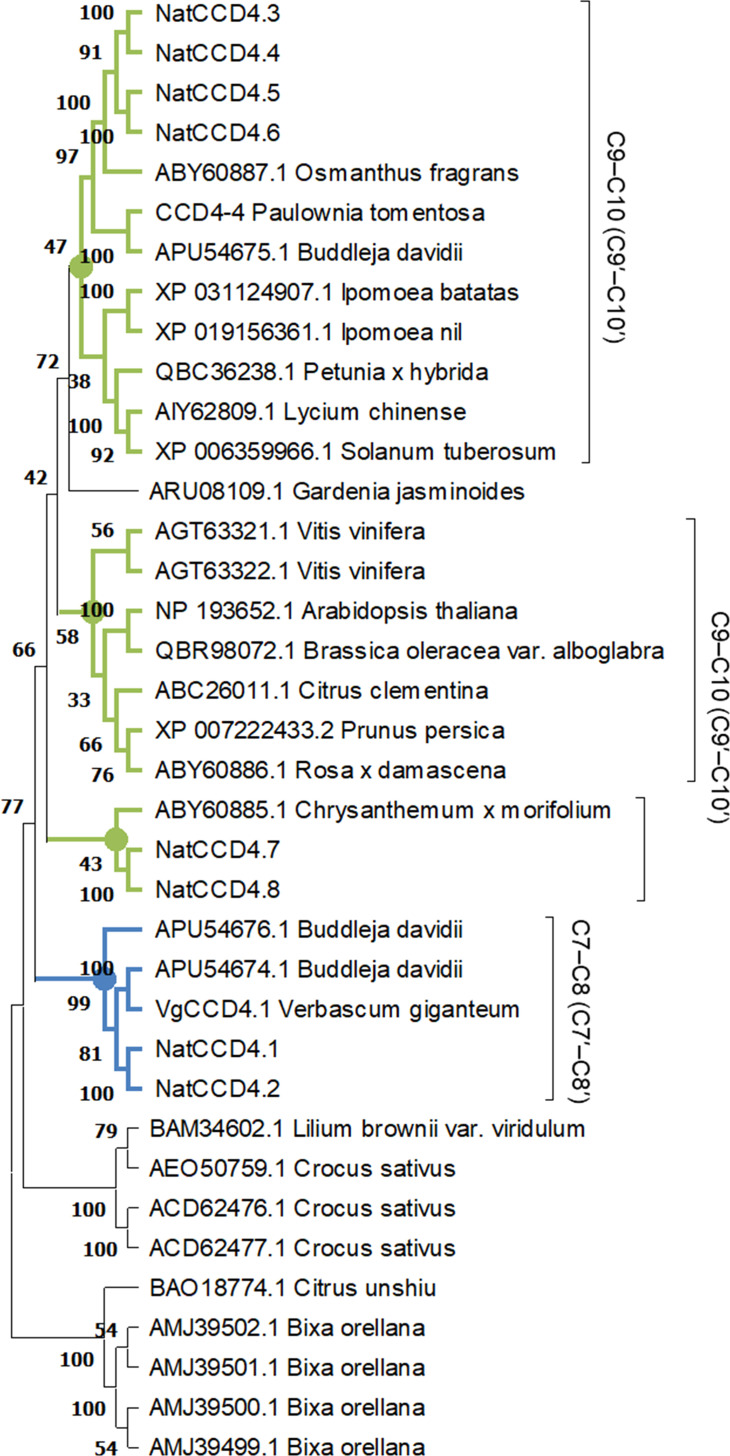
Phylogenetic relationship of amino acid sequences of CCD4 enzymes from different plant species. The phylogenetic tree was constructed by the maximum-likelihood method (https://megasoftware.net/) and bootstrap tests replicated 5000 times. The number on the nodes corresponds to the percentage of bootstrap values. The sub-tree including the CCD4 sequence with a C7-C8:C7′-C8′ cleavage activity is labelled in blue, while in green are labelled the cluster grouping enzymes which showed a C9-C10:C9′-C10′ activity. The activities reported for each CCD are shown on the right.

A phylogenetic tree with the identified CCDs and CCD protein sequences from *Arabidopsis thaliana* was inferred using the maximum-likelihood method (**Figure 3**). The CCD4 enzymes were closely related to each other, and these sequences were used to build an additional phylogenetic tree with those CCD4 sequences with characterized CCD activity in the literature (**Figure 4**). The CCD4 enzymes from *N. arbor-tristis* were placed in different sub-clusters. One of the subclusters containing 6 of the CCD4 enzymes identified in *N. arbor-tristis* was closely related to the cluster of CCD4 with a C9–C10 (C9′–C10′) cleavage activity, while the NatCCD4.1 and NatCCD4.2 were closely related to CCD4 enzymes from *Buddleja* and *Verbascum* which showed a C7–C8 (C7′–C8′) cleavage activity (**Figure 4**). This result suggested its involvement in the biosynthesis of crocins in *N. arbor-tristis*. Both genes were present in tandem in the analyzed genomic contig (NycArb205559), in the same orientation, separated by less than 2600 bp. However, contigs for NatCCD4.2 were not present in the flower transcriptome, suggesting the lack or reduced expression of this gene in this tissue. In fact, analysis of the promoter sequences of both genes showed a 48.06% identity (**Supplementary Figure S3**) suggesting a differential regulation.

Both proteins, which share 68.62% identity, showed the common characteristic seven-bladed β-propeller structure (**Supplementary Figure S4**) with a series of loops in whose center a Fe^2+^is localized. This cation is essential for the catalytic activity, ensuring the activation of oxygen for cleavage of carotenoid/apocarotenoid substrates (Harrison and Bugg, 2014). Around this divalent cation, four conserved histidine (H) residues form the first coordinated sphere while three glutamic acid (E) residues form the second coordinated sphere (**Supplementary Figure S4**).

### Functional characterization of CCD4 in *E. coli* cells

**Figure 5:**
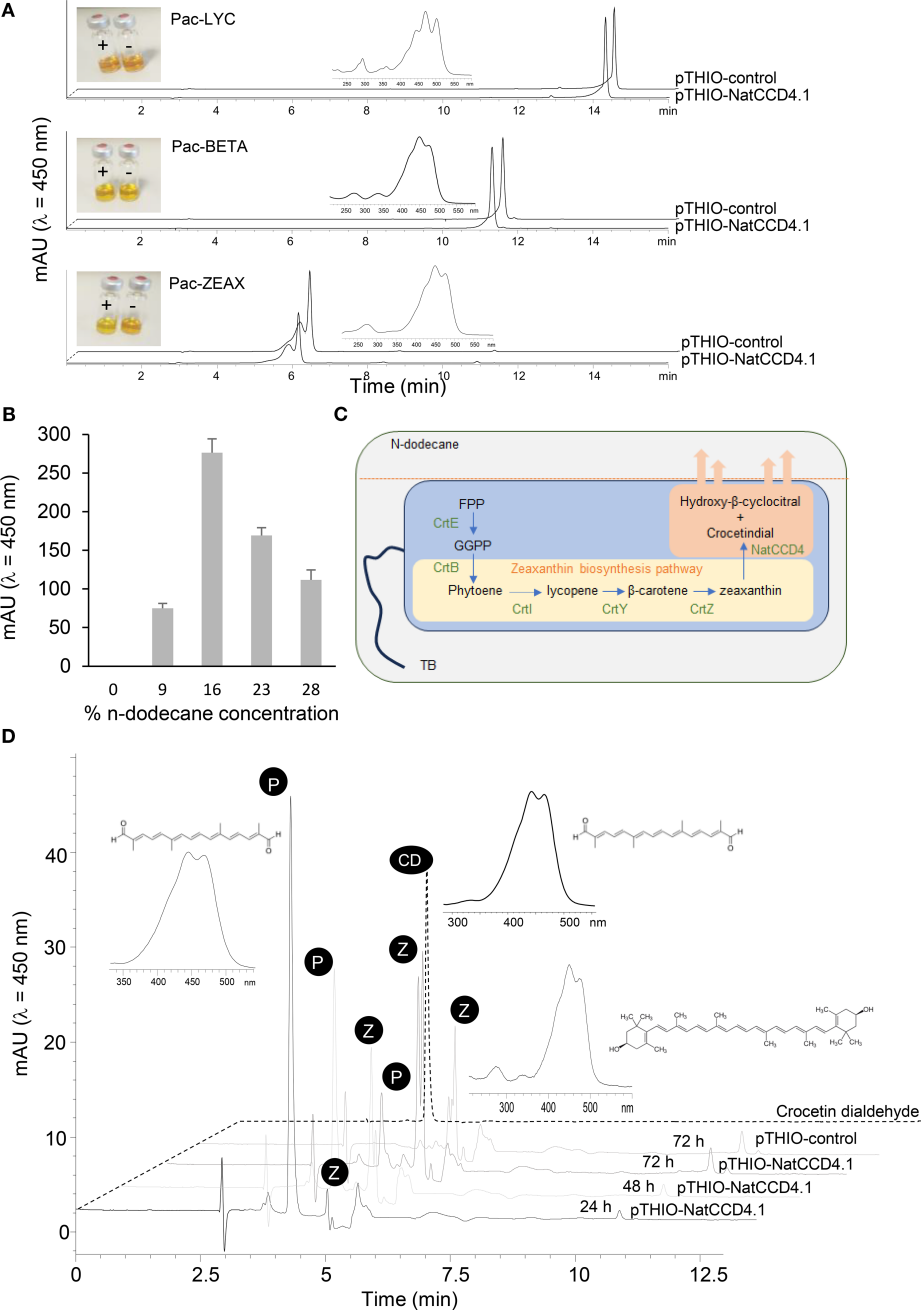
*In vivo* assays of NatCCD4.1 using *E. coli* cells accumulating different carotenoid substrates. **(A)** Representative HPLC-DAD chromatograms of apolar extracts obtained from *E. coli* cells expressing NatCCD4.1 and control cells. Insets depict the UV/vis spectra of lycopene, from the extracts of cells containing the Pac-LYC plasmid, spectra of β-carotene, from the extracts of cells containing the Pac-BETA plasmid, and the spectra of zeaxanthin, from the extracts of cells containing the Pac-ZEAX plasmid. Also, the colors of the apolar extracts are shown. **(B)** Quantification of crocetin dialdehyde obtained from *E. coli* cells producing zeaxanthin and expressing NatCCD4.1, after using different final concentrations of n-dodecane for the determination of NatCCD4.1 activity using an *in situ* two-phase culture system. **(C)** Schematic representation of the *in situ* two-phase culture system used for the detection of NatCCD4.1 reaction product. **(D)** Representative chromatogram profiles obtained in the HPLC-DAD analyses of NatCCD4.1 activity on zeaxanthin using the *in situ* two-phase culture system, with 16% n-dodecane final concentration and collection at different time points after arabinose induction. Insets are shown the structures and UV/vis spectra of crocetin dialdehyde (indicated by P) and zeaxanthin (indicated by Z).

To determine the activity of NatCCD4.1, the corresponding cDNA was cloned in the expression vector pTHIO-DAN1, affording arabinose-inducible expression. *E. coli* cells that co-express different carotenoid biosynthetic genes, allowing the synthesis of lycopene, β-carotene, or zeaxanthin, as described previously (Gomez-Gomez et al., 2020) were transformed. After induction of NatCCD4.1 expression with arabinose, carotenoids and apocarotenoids were extracted and analyzed by HPLC-DAD. Cleavage of carotenoid substrates in *E. coli* destroys the chromophore, causing a loss of color (also referred to as bleaching) (**Figure 5A**). The reduced levels of zeaxanthin in the cells expressing NatCCD4.1 suggested the cleavage of this substrate (**Figure 5A**). However, under the tested conditions, the detection of the carotenoid cleavage product from culture media and organic extracts of cell pellets from bleached *E. coli* cultures was not possible, suggesting that the aldehyde cleavage products were further modified or quickly degraded in *E. coli*.

### Two-phase culture using n-dodecane for *in situ* extraction of crocetin dialdehyde

To prevent apocarotenoid degradation in *E. coli*, a two-phase culture system using n-dodecane was performed for *in situ* extraction of apocarotenoids from the bacterial cells. This solvent has been previously used for the extraction of hydrophobic retinoids in *E. coli* (Jang et al., 2011), which significantly enhanced yield. Different final % concentrations of n-dodecane were tested to determine the optimal ratio for crocetin production. The final fractions of n-dodecane tested were 9%, 16%, 23% and 28%. The *in situ* extraction by dodecane could minimize intracellular degradation of the retinoids. Apocarotenoids are sequestered and more stable in the n-dodecane phase (**Figures 5B, C**), and could not be detected in the cell mass, where the substrate zeaxanthin was retained (**Supplementary Figure S5A** and **Figure 5C**). As a result, apocarotenoid production was measured only from the n-dodecane phase. The best results were obtained with 16% final mix after 48 h of growth (**Figure 5B**). Further, we tested different growth times using a final 16% n-dodecane mix to determine the impact on the production of crocetin dialdehyde, from 24, 48 and 72 h. The best result was obtained after 24 h of incubation (**Figure 5D**), with a 109.20 ± 3.23 mg/L production of crocetin dialdehyde, compared to the 61.00 ± 1.90 mg/L and 23.60 ± 1.12 mg/L obtained after 48 and 72 h, respectively (**Figure 5D**). In addition, longer incubation times resulted in the extraction of more carotenoid substrate in the organic phase, which is mainly present in the cell pellet (**Supplementary Figure S5B**), due to cell death and lysis. In addition, the obtained results are in agreement with previous observations in *E. coli* cells expressing *CsCCD2* (Lee et al., 2024), which showed that the decrease in crocetin dialdehyde accumulation after 27 h was related to the changes in the expression levels of *CsCCD2* mRNA, suggesting that *CsCCD2* mRNA expression/stability could be a potential target for increasing the yield of crocetin dialdehyde biosynthesis in bacteria. However, the decline in crocetin dialdehyde yield after 24 h of culture could be also due to additional factors, such as *E. coli* transitions into stationary phase, nutrient depletion, accumulation of metabolic byproducts, and decreased cell viability can negatively affect protein production, as observed in previous studies (Liu et al., 2020c; Ruan and Bourne, 2024). Additionally, the catabolism of arabinose, the inducer used for expression, reduces its availability, further diminishing protein synthesis, a phenomenon also reported in previous research (Siegele and Hu, 1997). Thus, the observed decline in product yield is likely the result of a combination of mRNA stability issues, metabolic shifts, and resource depletion, as seen in other recombinant *E. coli* expression systems (Rosano and Ceccarelli, 2014).

Once the optimal conditions were established for the two-phase culture system for the *in situ* extraction of the apocarotenoids products, we decided to test the activity of CsCCD2 using *E. coli* cells accumulating zeaxanthin (**Supplementary Figure S6A**), and NatCCD4.2 using *E. coli* cells accumulating lycopene, β-carotene, and zeaxanthin (**Supplementary Figure S6B**). Under the experimental conditions tested, bacterial cells accumulating zeaxanthin and expressing CsCCD2 exhibited lower crocetin dialdehyde production compared to cells expressing NatCCD4.1 (**Supplementary Figure S6A**). However, no products were detected in cells expressing NatCCD4.2 when using any of the three carotenoid substrates under these optimized conditions (**Supplementary Figure S6B**).

### Functional characterization of CCD4 in *N. benthamiana*

**Figure 6:**
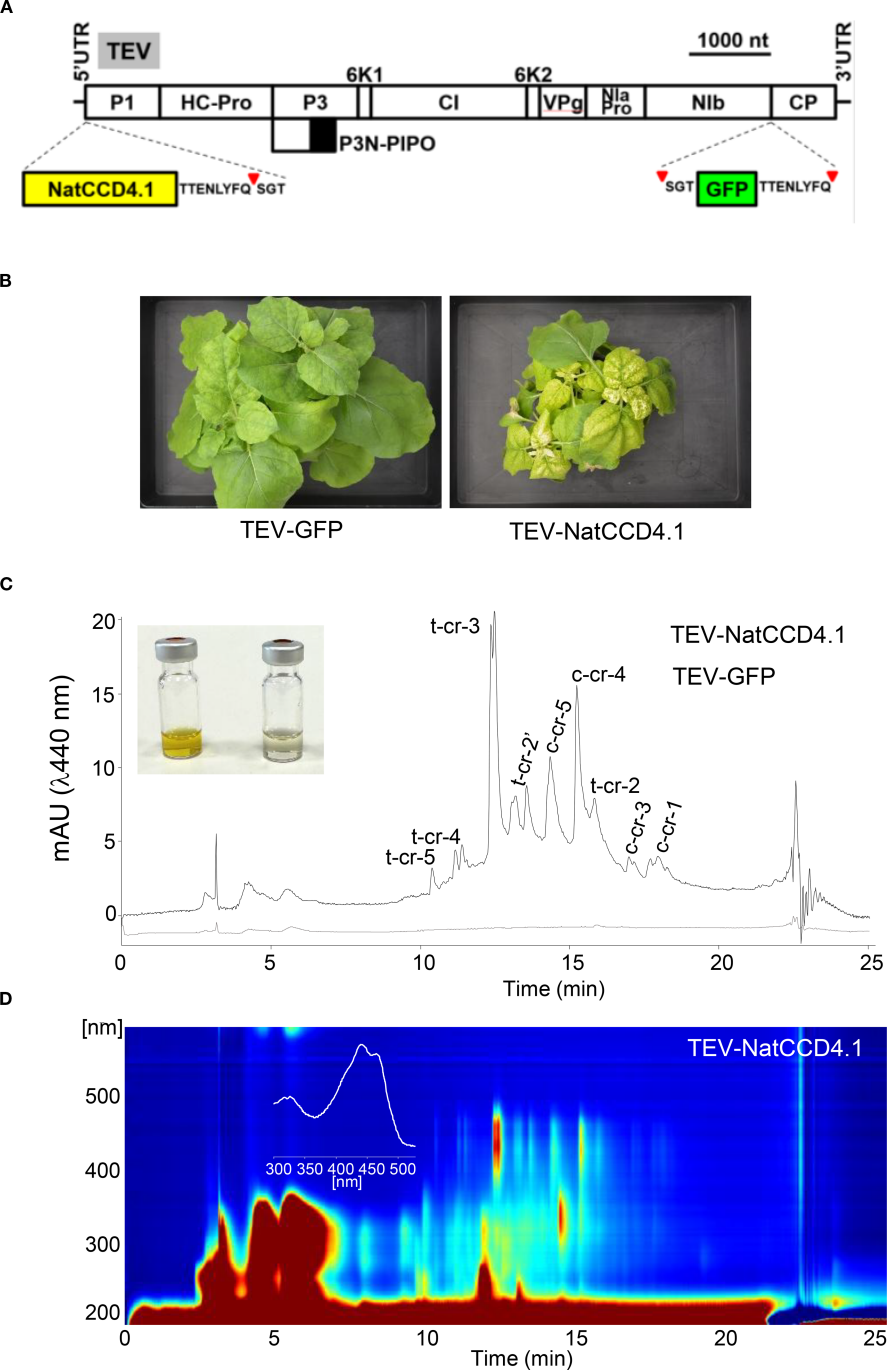
Crocin production in *N. benthamiana* plants using a TEV recombinant clone that expresses NatCCD4.1. **(A)** Schematic representation of TEV genome indicating the position where the GFP (green box) or NatCCD4.1 (yellow box) were inserted. The sequence of the artificial NIaPro cleavage site to mediate the release of the recombinant proteins from the viral polyprotein is indicated. The red arrow points the cleavage position. Lines represent TEV 5’ and 3’ UTR and boxes represent P1, HC-Pro, P3, P3N-PIPO, 6K1, CI, 6K2, VPg, NIaPro, NIb and CP cistrons, as indicated. Scale bar corresponds to 1000 nt. **(B)** Pictures of representative leaves from plants mock-inoculated and agroinoculated with TEV-GFP and TEV-NatCCD4.1, as indicated, taken at 14 dpi. Scale bars correspond to 5 mm. **(C)** Representative chromatographic profile run on an HPLC-DAD/UV system and detected at 440 nm of the polar extracts of tissues infected with TEV-GFP, and TEV-NatCCD4.1. Peak abbreviations correspond to: c-cr, cis-crocetin; t-cr, trans-crocetin; c-cr1, cis-crocin 1; t-cr1, trans-crocin 1; t-cr2, trans-crocin 2; t-cr2′, trans-crocin 2′; c-cr3, cis-crocin 3, t-cr3, trans-crocin 3; c-cr4, cis-crocin 4; t-cr4, trans-crocin 4; c-cr5, cis-crocin 5; t-cr5, trans-crocetin 5. **(D)** HPLC-DAD/UV isoplot chromatogram of polar extracts and absorbance spectra of the major crocin detected in the polar extracts of *N. benthamiana* tissues infected with TEVΔNIb-NatCCD4.1. Analyses were performed at 13 dpi.

Subsequently, we constructed a recombinant virus (**Figure 6A**) to express NatCCD4.1 (TEV-NatCCD4.1) in *N. benthamiana* plants. NatCCD4.1 was predicted to be localized in the plastid and must contain the native amino-terminal transit peptide to target the enzyme to the plastids. NatCCD4.1 was inserted in the virus genome in a position that correspond to the amino terminal end of the viral polyprotein (Martí et al., 2020). In addition, at the 3′ end of the cloning site, the construct contains a sequence for an artificial NIaPro cleavage site to allow the release of NatCCD4.1 from the viral polyprotein. Based on our previous work, the -8/+3 site that splits NIb and CP in TEV was used. The obtained recombinant construct TEV-NatCCD4.1 was introduced in *A. tumefaciens*, and positive clones were agroinoculated in *N. benthamiana* plants. TEV-GFP that expresses GFP, between viral NIb and CP cistrons, was used as a control. Symptoms of infection were observed approximately 8 dpi in all plants agroinoculated with TEV-NatCCD4.1 and TEV-GFP. A unique yellow pigmentation was observed in tissues of plants agroinoculated with TEV-NatCCD4.1 at approximately 14 dpi (**Figure 6B**). Symptomatic leaves from plants infected with TEV-GFP, and TEV-NatCCD4.1 were collected and subjected to extraction and analysis to determine their crocin and carotenoid profiles. Analysis of the polar fraction of leaves infected with TEV-NatCCD4.1 showed a series of peaks with maximum absorbance around 440 nm, corresponding to crocins with different degrees of glucosylation (**Figures 6C, D**). These peaks were absent from the extracts from tissues from mock-inoculated control plants or plants infected with TEV-GFP. Subsequently, the levels of carotenoids and chlorophylls in the apolar fractions were also investigated (**Supplementary Figure S7**). The comparison of the profiles of apolar extracts from tissues infected with TEV-GFP and TEV-NatCCD4.1 showed reduced levels of chlorophyll and pheophytin in TEV-NatCCD4.1. At the carotenoid level, major differences were observed in the level of zeaxanthin, with a 96.61% reduction, followed by an 87.71% reduction of β-carotene, and 75.70% reduction of lutein levels in the leaves of TEV-NatCCD4.1-infected plants (**Supplementary Figure S7**), which further reinforced the additional activity of this enzyme in targeting zeaxanthin. Overall, these results revealed a remarkable accumulation of 2.32 ± 0.69 mg/g of crocins in *N. benthamiana* dry weight (DW) leaf tissue.

## Discussion

Crocins have a wide range of biological properties and represent valuable metabolites with multiple applications in different industrial sectors as pharmaceuticals, nutrients, and cosmetic ingredients (Guo et al., 2021; Ali et al., 2022). Current commercial production of crocins is based on purification from saffron stigmas and gardenia fruits. In addition, crocin biosynthesis has been evaluated in bacteria (Pu et al., 2020; Lee et al., 2024), yeast (Liu et al., 2020a), and plants using the CCD enzymes from saffron, gardenia, *Bixa*, *Buddleja*, and *Verbascum* (Gómez-Gómez et al., 2023; Morote et al., 2024). Here, we report the production of crocetin in *E. coli* and crocins in *N. benthamiana* using a CCD4 enzyme identified in *N. arbor-tristis*. The analysis of the genome of *N. arbor-tristis* allowed the identification of two CCD4 genes, which shared 68.62% identity, encoding for CCD4 enzymes that cluster with previous CCD4 enzymes involved in crocetin biosynthesis in *Verbascum* (Morote et al., 2024) and *Buddleja* (Ahrazem et al., 2017). However, only one of these two proteins, NatCCD4.1, was active for crocetin dialdehyde biosynthesis. All these three plants, *N. arbor-tristis*, *Buddleja*, and *Verbascum*, belong to the Scrophulariaceae family, which suggests that the specialization of these enzymes to produce crocetin dialdehyde occurred before the divergence of these three species. As for the case of the enzymes characterized in *Verbascum* and *Buddleja*, NatCCD4.1 showed high specificity for zeaxanthin, which is in agreement with the detection of safranal among the volatiles emitted by the flowers of *N. arbor-tristis* (Siriwardena and Arambewela, 2014; Naik et al., 2016), *Buddleja* and *Verbascum* (Chen et al., 2012; Morote et al., 2024). However, the enzyme from *Verbascum* also recognizes β-carotene as substrate (Morote et al., 2024). In *Verbascum*, crocins are homogenously distributed in the petals of the flower, which showed a yellow coloration, while in *N. arbor-tristis* and *Buddleja* the accumulation of crocins is specifically restricted to the calix (**Supplementary Figure S8**), which can serve as visual cues for pollinators, enhancing the success of plant pollination.

Apocarotenoids are, in general, highly sensitive to light, temperature, and oxygen (Wijesekara and Xu, 2024), and crocetin is not an exception (Carmona et al., 2006; Liu et al., 2023). Therefore, apocarotenoids produced in heterologous microbial hosts are often vulnerable to degradation (Han and Lee, 2021). To reduce the intracellular degradation of crocetin dialdehyde, and to solve the problem of limited solubility, a two-phase *in situ* extraction using n-dodecane has been applied for retinoid production in *E. coli* and *S. cerevisiae* (Jang et al., 2014; Sun et al., 2019). This strategy was used in this study to improve the detection of crocetin dialdehyde in the bacterial assay. By using this methodology, crocetin dialdehyde was recovered in the organic phase 24 h after the induction of NatCCD4.1 expression in the bacterial cultures, with low levels of zeaxanthin detected in this phase. Therefore, this methodology allowed efficient recovery of crocetin-dialdehyde without the necessity of additional high-cost processing steps such as sequential cell-disruption and purification of the pigments from the cell pellet using organic solvents. In addition, the obtained yield of crocetin dialdehyde was higher than those levels previously reported in *E. coli* using the CsCCD2 enzyme from saffron, 5.14 mg/L (Lee et al., 2024), 4.24 mg/L (Wang et al., 2019) and 34.77 ± 1.03 mg/L (Lee et al., 2024), and also above of the levels obtained using yeast; the expression of *CsCCD2* in this host produced 1.21 mg/L (Chai et al., 2017). Further optimization of the system allowed the obtention of 1.95 mg/L of crocetin (Song et al., 2020). In another strategy developed in yeast, using a temperature-responsive crocetin-producing strain and increasing the copy number of *CsCCD2* gene using the CRISPR-Cas9 based multiplex genome integration technology, 1.05 mg/L were produced, suggesting the existence of different bottleneck points for crocetin production in yeast (Liu et al., 2020a). In any case, the industrial potential of *E. coli* as a sustainable platform for crocetin-dialdehyde production is endorsed by the inexpensive starting material, allowing a one-step process that has significant advantages compared with the currently used methodology for the obtention of crocetin-dialdehyde from saffron or gardenia extracts using different chemicals and processes needed to convert crocins into crocetin dialdehyde (Guo et al., 2021). In addition, the rapid growth and low growth requirements contribute to the fast production of this metabolite at reduced cost.

The transient expression system using *N. benthamiana* has become a preferred plant-based platform due to its advantage in high yield metabolites production and speed, as well as the lack of concern about transgene escape or contamination of food crops (Nosaki et al., 2021). Virus-driven expression of NatCCD4.1 in *N. benthamiana* plants resulted in the accumulation of remarkable amounts of 2.32 ± 0.69 mg of crocins per gram (DW) of infected tissues in only 14 dpi. Previous experiments using this system and different CCDs from species accumulating crocins showed variable levels of crocins accumulation in leaves. The CCDs previously tested include BdCCD4.1 (0.75 ± 0.02 mg/g DW), CsCCD2L (2.18 ± 0.33 mg/g DW), and VgCCD4.1 (1.78 ± 0.11 mg/g DW) (Martí et al., 2020; Morote et al., 2024). These findings show that expression of a single CCD gene can consistently result in crocin accumulation at mg/g DW levels in *N. benthamiana* tissues. This efficiency highlights the advantage of plant hosts over microbial systems such as yeast or bacteria, where multiple transgenes encoding carotenoid substrates, aldehyde dehydrogenases, and glucosyltransferases are required (Song et al., 2020; Lee et al., 2024). In plants, crocin biosynthesis relies on endogenous metabolic capacity. Carotenoid substrates are synthesized in the plastids, where NatCCD4.1 cleaves zeaxanthin to produce crocetin dialdehyde. This intermediate is then oxidized by aldehyde dehydrogenases and glycosylated by UDP-glucosyltransferases in the cytosol, and the resulting crocins are ultimately stored in the vacuoles of leaf tissues (Diretto et al., 2019; Liu et al., 2020b; Morote et al., 2025). Nevertheless, the use of viral vectors for transient expression can impose physiological costs on the host plant. Viral infection may activate defense responses, induce stress, and shorten leaf lifespan, which in turn can affect metabolite yield and profiles. Even so, these effects are generally less severe than the metabolic imbalances and chronic stress observed under constitutive CsCCD2L expression, which has been associated with reduced crocin accumulation (Ahrazem et al., 2022; Huang et al., 2022).

## Conclusions

Significant levels of crocetin dialdehyde and crocins have been produced in this study by the expression of a unique identified gene in *N. arbor-tristis*, NatCCD4.1, in bacteria and in planta, respectively. The obtained levels are in the order of mg/L and mg/g (DW), without the introduction of further optimization procedures that can enhance the current crocetin titter in *E. coli* and the crocins content in *N. benthamiana*. Improvements could be obtained from development of fermentation processes (such as fed-batch cultivation), or media formulations, in the case of crocetin production in *E. coli*, or the introduction of additional genes that could enhance the levels of precursor in the case of *N. benthamiana*.

## Data Availability

The datasets presented in this study can be found in online repositories. The names of the repository/repositories and accession number(s) can be found in the article/**Supplementary Material**.
