# Supplementary material for: Biotechnological production of crocetin and crocins using a carotenoid cleavage dioxygenase (CCD4) from Nyctanthes arbor-tristis
Source: Front Plant Sci. 2025 Oct 17;16:1671592. doi: 10.3389/fpls.2025.1671592 (PMC12575119; doi:10.3389/fpls.2025.1671592)
Supplement: Supplementary file 1 [file DataSheet1.pdf]

Supplementary Data

Biotechnological Production of Crocetin and Crocins Using a Carotenoid Cleavage Dioxygenase (CCD4) from Nyctanthes arbor-tristis

Lucía Morote[1], Elena Moreno Giménez[1], Ángela Rubio-Moraga[1,2], Alberto José López Jiménez[1,2], Verónica Aragonés[4], Oussama Ahrazem[1,2], José-Antonio Daròs[4], Lourdes Gómez-Gómez[1,3,*]

[1]Instituto Botánico. Departamento de Ciencia y Tecnología Agroforestal y Genética. Universidad de Castilla-La Mancha, Campus Universitario s/n, 02071 Albacete, Spain.
[2]Escuela Técnica Superior de Ingenieros Agrónomos y de Montes y Biotecnología. Departamento de Ciencia y Tecnología Agroforestal y Genética. Campus Universitario s/n, 02071 Albacete, Spain.
[3]Facultad de Farmacia, Campus Universitario s/n, 02071 Albacete, Spain.
[4]Instituto de Biología Molecular y Celular de Plantas (Consejo Superior de Investigaciones Científicas-Universitat Politècnica de València), 46022 Valencia, Spain.

[*]Corresponding author: Lourdes Gómez Gómez
E-mail: marialourdes.gomez@uclm.es

Fig. S1

A

```
>NatCCD4.1
MTSMGTLSSSSSLLVNLHSKASSPPKTYKFRVSSLRVEENHETSILPEKAFSSRTTLQKLVQLIGKSTPRKEVVKKKKEPSLVATILNAYEDFICTFLDL
PLPPSLDPKHVLSGNYAAVDELPPTPCEVVEGALPSCLDGAYLRNGPNPQFIPRGPYHLFDGDGMLHVIKISQGKAVFCSRYVKTHKYIIEHKNGSPVIP
SIFSSFNGFSATMARLVPFVVRSLAGQFNPSTHGIGVANTSLALIGGDLFALAESDLPYKLKLTPDGDVITQGRHTFCSKPVMSMTAHPKTDPNTGEAFG
FAYNVFHPFLTYFRINREGVKQKDIPINSLTRSSFIHDFAVTKNYVVFPETQIVIDPMNIIRGKSPVGTDLAQVPRLGILPRYAENDAEMTWIDAPGLNM
VHAFNAWEEDDGNRVVILASNAFEFESPSDPMDFLHLSVDRVEINLKEKKVLRQPLCSTNLDLAVINPSYAGKKNRFIYAAVTTTTPKRAGVMKLDLSLF
EADNRDCAVGSRLYEPGCFGGEPFFVPRDPGSREDVDEDDGYLVTYLHNEKTGESRFLVMDAKSPELEIVAQVKLPGRVPYGFHGLFVPESELKDM
>NatCCD4.2
MGTLSSSFLPQIPLHFHGKTPSPPNTAKIRVSSARRTLQKLEGITERRNTKKPTRQEQEQVVWKTKPSLLATIFNSFEDFISTFIDLPLHPSLDPKHVLS
GNFAPVDELPPTPCEVVEGALPSCLDGAYIRNGPNPQFIPHGPYHLFDGDGMLHCIRIFQGKAIFCSRYVKTYKYLIEHKNGSPVFPSVFSSFNGFFASM
ARCMLAVARVMAGQFSPMIHGFGLANTSIALFGGYLFALGESDLPYHINLTSNGDIITLGRHAFYSKPFLNMTAHPKLDPNTGELFAFAYNIYHPFLTYF
RINKEGVKQNDMLINSLKRSSFIHDFAVTKNYAIFPDIQIMIQPMDIMRGRSPVGVDPAKAPRLGVLPRYAKNEIEMSWMDVPGFNMLHAVNAWEEDDGG
KIVIVASNILHVEHALDRMDLIHLSLEKVEIKVKEKKILRQSVSSKSLDFGVINPAYVGKKNRYVYAAMTDPMPKIAGVLKLDLSLLEADKGNCTVGSRL
YESGCYGGEPFFVQRDPSNPDAEEDDGYLVTYMHNENTGESMFLVMDAKFPDLHIVAKVKLPGRVPYGFHGLFVPESELKNLRNFHN
>NatCCD4.3
MDTLSSSFLPKLHLKYFLPPSLKSARPTFLSVSSVRTEDKPQTTTTNGTAGREVAQPLKKETPPLPNPREILPSFLAPKKMESLLSPSTIFNVVDNFINT
CIDPPLHPSTDPKYVFLNNYAPVDELPPTQCQVVEGTLPTCLNGAYIRNGPNPQFFPRGPYHTFEGDGMLHSIRIFNGKATFCSRYVKTYRYIVENKNGF
PIFPNVFSGFNGLTALATRGALAAARILAGQFNPANGTGPANTSLALVGGKLYALGESDLPYTVKVAEDGDIITQGRHDFDGKLIMSMTAHPKVDPDTKE
AFAFRYGAISPFLTFFRINPDGTKQPDVPIFSMKSPSLIHDFAITKKYAIFPEIQIGINPLKMMAGGSLIGTNPGKVPRLGVIPRYAKDESEMKWFDVPG
FNILHAINSWEEDDGDTIVLVAPNILSVEHTLERVDLIHLSVEKVKIDLKTGMVSRIPLSTRNLDFGAINPSYTGKKNKYVYAAIGDPMPKIPGVVKLDI
SVSGSDHRDCIVASRLFGEGCLGGEPFFVAKEPDNPSADEDDGYVVSYVHNEKSGESSFLVMDAKSPNLDIVAAVKLPSRVPCGFHGLFVREDDLKKL
>NatCCD4.4
MDTLSSSFLPKLHLKYFLPPSLKSARPTFLSVSSVRTEDKPQTTTTNGTAGREVAQPLKKETPPLPNPREILPSFLAPKKMESLLSPSTIFNVVDNFINT
CIDPPLHPSTDPKYVFLNNYAPVDELPPTQCQVVEGTLPTCLNGAYIRNGPNPQFFPRGPYHTFEGDGMLHSIRIFNGKATFCSRYVKTYRYIVENKNGF
PIFPNVFSGFNGLTALATRGALAAARILAGQFNPANGTGPANTSLALVGGKLYALGESDLPYTVKVAEDGDIITQGRHDFDGKLIMSMTAHPKVDPDTKE
AFAFRYGAISPFLTFFRINPDGTKQPDVPIFSMKSPSLIHDFAITKKYAIFPEIQIGINPLKMMAGGSLIGTNPGKVPRLGVIPRYAKDESEMKWFDVPG
FNILHAINSWEEDDGDTIVLVAPNILSVEHTLERVDLIHLSVEKVKIDLKTGMVSRIPLSTRNLDFGAINPSYTGKKNKYVYAAIGDPMPKIPGVVKLDI
SVSGSDHRDCIVASRLFGEGCLGGEPFFVAKEPDNPSADEDDGYVVTYVHNEKNGESSFLVMDAKSPNLDTVAAVKLPRRVPYGFHGIFVRENDLNKL
>NatCCD4.5
MDTLSSPFLPKLHPKFIWSPPLKSPHPSCLSISSVRIEDKPQTTTTGGREVAQPLKKETPPPSSPRKIRSSTLSTTRPSLEPFSFPSTIFNVVDNFINTF
VDPPLRPSVDPRYVLCDNFAPVGELPPTQCLLVEGSLPASLNGAYIRNGPNPQFLPRGPYHLFDGDGMLHSIRISDGKATLCSRYVKTYRYTVENERGYP
VFPNVFSGFNGLRASAARGAVTAARVLAGEFNPINGAGLANTSLALIAEKLYALGESDLPYEVKVAPDGDIITHGRHDFDGKLFMSMTAHPKVDLDTGEA
FAFRYGPMPPFLTFFRIDPDGTKQPDVPIFSMTSPSFMHDFAITKKYAIFPEIQIGMNPMEMMAGGAPVGANPGKVPRLGVIPRYAKDESEMKWFDVPGF
NIIHAINSWEEDDGDTIVLVAPNILSVEHTLERMDLIHASVEKVKIDLKTGMVSRSPISTRNLDFGAINPAYTGKKNKYVYAAIGDPMPKISGLVKLDVS
VSDSDRRDCIIASRLFGEGCFGGEPFFVAREPNNPNADEDDGYVVTYVHNEKNGESSFLVMDAKSPNLDTVAAVKLPRRVPYGFHGIFVRENDLNKL
>NatCCD4.6
MDTLSSSFLPKLHPKFILSPPLKSPHPTCLSISSVRIEDKPQITTTTTSGRREVARPLKKETPSPPSSPRKIRSSTLSTMRPSLEPFSFPSTIFNVVDNF
INTFVDPPLRPSVDPRYVLCDNFAPVGELPPTQCLVVEGSLPTSLNGAYIRNGPNPQFLPRGPYHLFDGDGMLHSIRILDGKATLCSRYVKTYRYNVENE
RGYPVFPNVISGFNGLTASAARGAVTAARVLAGQFNPTNGVGLANTSLALIGGKLYALGESDLPYEVKVAPDGDIITHGRHDFDGKLFMSMTAHPKMDLD
TGEAFAFRYGPMPQIVTFFRIDPDGTKQPDVPIFSMTSPSFMHDFAITKKYAIFPEIQIGMNPMEMMAGGAPVGANPGKVPRLGVIPRYAKDESEMKWFD
VPGFNIIHAINSWEADDGDTIVLVAPNILSVEHTLERMDLIHASVEKVKIDLKTGMVSRSPISTRNLDFGVINPAYTGKKNKY
>NatCCD4.7
MSSSIHTPNSLHDSSIRIDISSTDNAISRSESNTSLIFNALDEFINKFIDPPRRPSVNPRHVLSGNFAPVDELSPTECEILEGFLPPCLDGVYVRNGPNP
QFFPQGPHHLFDGDGMLHSIKISQGKATFCSRYVKTYRYMLEREMGSSIIPHVFSSFNGLVACLARGVVSAARMLTGQFTHVNGLGVASTSLAFFGGNLY
ALDESDLPYAIRMAQDGDIITLGRCDFNGKLVTNMTAHPKTDQDSGETFAFRYSFMRPYLSFFRFDNDGIKQPDVPIFSMASPSFVHDFAITKNYAIFPQ
IQLEVRPLNMVFGSGSPLRVDQSRNPRIGVIPRYAKDETEIKWFKVPGLNILHAINAWEENSGDTIVMVAPNILTLEHFIERMDLFHSLIEKVKIDLKTG
TVSRHPLSTRNLEFGVINPAYVGKKNKYVYAGVEDLMPKTAGVVKLDITSMKCRDCVVASRFYGHGCYGGEPLFVAKEPNNPNADEDDGYLVTYVHDENT
GNSRFLVMDAKSATLEIVTAVTLPQRVPYGFHGLFVREEEL
>NatCCD4.8
MDAISSHLLPKLSHHQKFTCFHMPKRKPSSSHLPNSLNVSSIRIDASSSSSSTSTSAATDDVTTATTSSAAHLLKKQAPSRLKINTSMIFDALDEFITKF
IDPPLRPSTDPRYVLSGNFAPVDELSPTECEVLDGSLPSCLDGVYIRNGPNPQFLPSGPYHSFDGDGMLHSIKISQGKATFCSRYVKTYRYMLEHEMGSK
VIPHVFSGFNSLMACVARGTVSAARMLTGQYTHTSGIGLANTSVAFFGGSLYALGESDLPYAIKVTQEGDIITLGRCDFNGKLVTNMTAHPKTDPDTGET
FAFRYSAIRPFLTFFRFDANGKKQDDVPIFSMTSPSFVHDFAITKNYAIFPEIQLGMRPLDVIFGLGPLIGMDWSKVPRLGVITRYAKDETETKWFNVPG
FNILHAINAWEENGGDTIVLVAPNILRLEHCIERMDLIHSSIEKVKIDLKTRVVSRHPLSTRNLEFGVINPTYLGMKNRYVYASIGDPMPKVSGVVKLDV
SLSGQDSHDCVVASRLYESGCYGGEPFFVAKEQNNPNLDEDDGYLVSYVHNENTKESTFLVMDAKSATLEIIAAVNLPQRVPYGFHGLFGKTYSMIESQN
NTSEINVEGDPTHSKGPNNESVVLARR
```

B

Percent Identity Matrix - created by Clustal2.1

1: NatCCD4.1	100.00	68.62	55.65	55.48	54.31	46.46	53.90	51.99
2: NatCCD4.2	68.62	100.00	59.83	59.66	59.03	51.50	57.25	56.55
3: NatCCD4.3	55.65	59.83	100.00	98.66	80.98	71.27	63.89	62.86
4: NatCCD4.4	55.48	59.66	98.66	100.00	82.32	71.27	63.89	62.52
5: NatCCD4.5	54.31	59.03	80.98	82.32	100.00	84.52	64.63	63.37
6: NatCCD4.6	46.46	51.50	71.27	71.27	84.52	100.00	57.58	56.64
7: NatCCD4.7	53.90	57.25	63.89	63.89	64.63	57.58	100.00	76.52
8: NatCCD4.8	51.99	56.55	62.86	62.52	63.37	56.64	76.52	100.00

Fig. S1. CCD4 sequences identified *in N. arbor-tristis*. A) Prediction of 8 amino acid sequences of CCD4 enzymes encoded by genes identified in the genome of *N. arbrt-tritis*. B) Percent identity matrix among the CCD4 enzymes created by Clustal2.1.

Fig. S2

NatCCD4.1

Prediction: Plastid, Soluble

Localization	Plastid	Cytoplasm	Mitochondrion	Endoplasmic reticulum	Nucleus	Peroxisome	Extracellular	Lysosome/Vacuole	Golgi apparatus	Cell membrane
Likelihood	0.9999	0.0001	0	0	0	0	0	0	0	0

Type	Soluble	Membrane
Likelihood	0.8686	0.1314

NatCCD4.2

Prediction: Plastid, Soluble

Localization	Plastid	Cytoplasm	Mitochondrion	Nucleus	Endoplasmic reticulum	Extracellular	Peroxisome	Lysosome/Vacuole	Golgi apparatus	Cell membrane
Likelihood	1	0	0	0	0	0	0	0	0	0

Type	Soluble	Membrane
Likelihood	0.9107	0.0893

NatCCD4.3

Prediction: Plastid, Soluble

Localization	Plastid	Cytoplasm	Mitochondrion	Nucleus	Endoplasmic reticulum	Extracellular	Peroxisome	Lysosome/Vacuole	Golgi apparatus	Cell membrane
Likelihood	1	0	0	0	0	0	0	0	0	0

Type	Soluble	Membrane
Likelihood	0.8869	0.1131

NatCCD4.4

Prediction: Plastid, Soluble

Localization	Plastid	Cytoplasm	Mitochondrion	Nucleus	Endoplasmic reticulum	Extracellular	Peroxisome	Lysosome/Vacuole	Golgi apparatus	Cell membrane
Likelihood	1	0	0	0	0	0	0	0	0	0

Type	Soluble	Membrane
Likelihood	0.8844	0.1156

NatCCD4.5

Prediction: Plastid, Soluble

Localization	Plastid	Cytoplasm	Mitochondrion	Nucleus	Endoplasmic reticulum	Extracellular	Peroxisome	Lysosome/Vacuole	Golgi apparatus	Cell membrane
Likelihood	1	0	0	0	0	0	0	0	0	0

Type	Soluble	Membrane
Likelihood	0.9003	0.0997

NatCCD4.6

Prediction: Plastid, Soluble

Localization	Plastid	Cytoplasm	Mitochondrion	Nucleus	Endoplasmic reticulum	Extracellular	Peroxisome	Lysosome/Vacuole	Golgi apparatus	Cell membrane
Likelihood	1	0	0	0	0	0	0	0	0	0

Type	Soluble	Membrane
Likelihood	0.905	0.095

NatCCD4.7

Prediction: Plastid, Soluble

Localization	Plastid	Mitochondrion	Cytoplasm	Nucleus	Extracellular	Peroxisome	Endoplasmic reticulum	Lysosome/Vacuole	Golgi apparatus	Cell membrane
Likelihood	0.9992	0.0004	0.0003	0	0	0	0	0	0	0

Type	Soluble	Membrane
Likelihood	0.8146	0.1854

NatCCD4.8

Prediction: Plastid, Soluble

Localization	Plastid	Mitochondrion	Cytoplasm	Nucleus	Endoplasmic reticulum	Extracellular	Lysosome/Vacuole	Peroxisome	Golgi apparatus	Cell membrane
Likelihood	1	0	0	0	0	0	0	0	0	0

Type	Soluble	Membrane
Likelihood	0.8953	0.1047

Fig. S2. Predicted in silico localization results of the different NatCCD4 enzymes using DeeoLoc (https://services.healthtech.dtu.dk/services/DeepLoc-1.0/).

Fig. S3

```
P-NatCCd4.2     tcgtcatttgcgaagaatgacccaagtaattaactgatattctggtagaatcttgaaaca     60
P-NatCCd4.1     ----------------------ca---agtaaaccgcaattctgtagtgattgtaatata     35
                                      **   * * *** *  ******     **  * * * *

P-NatCCd4.2     atgacggcaagtataatcgtcc--cgaataccagccaaagcacttatctccaaattttag     118
P-NatCCd4.1     --------cagttcaatggtttggctaagaccaactaaaacttatggattagaatcggcg     87
                         ***  *** **    * ** **** * *** *   *   *   ***    *

P-NatCCd4.2     ctcaattattttcatcttaattttcatgtcgtccgaagctagatgaatatt---------     169
P-NatCCd4.1     tttttttcctagaattgggtttctcctgtgtaaacagtgtagagaaataatgtggctaag     147
                 *   **  *   **      ** ** ***      *   ****  **** *

P-NatCCd4.2     attatct---------ttattataataataataataacgaca-----------ttgccga     209
P-NatCCd4.1     actagctaaaacttctggattagaatcggtgtgtaagcagtatagagaaataatgcctga     207
                * ** **           **** ***     *   * *   *           *  * **

P-NatCCd4.2     ttctac-------cttagtatcttctttacagttaaaatacatcttcatatcccaagaat     262
P-NatCCd4.1     caaaaactttggattccgccatttctttcaattttggcaaattcatcatctcaatagcta     267
                    *         *  *    ******  * **     *  ** **** **   **

P-NatCCd4.2     aaatattttgatgttatctttatgatctgttctgtcggctgcatttgttttattcatagt     322
P-NatCCd4.1     aaagagggca-----ctc--------cttgaagttcggctgcatgttt-----ccttcta     309
                *** *           **        **      ********** * *      * *

P-NatCCd4.2     tttggagatgctttaggtttgatagaaatacaaaatgtttgaaaatggaaacagaatact     382
P-NatCCd4.1     tttgggaccaaataataattcaaatgcctaataaatgttattatgttgagacttgagaga     369
                *****       * *   ** * *    **  *******   *  * ** **   * *

P-NatCCd4.2     atagaagggggtatcctgcaactggtaat-atatacattagtgcaaagacagatttactt     441
P-NatCCd4.1     agaaaaaatgttttagaaccaatggttggtttggtttttatcagtaagatgacttgactc     429
                * * **   * * *    * * ****     *     ***      ****    ** ***

P-NatCCd4.2     agttaaacgtagcaacaaaatcaccacccctaaaaatcatgttacatcaatacatcccac     501
P-NatCCd4.1     aataagaaaaaacatctattttctttaccattatatatgtcctcgatctattcatcccga     489
                * * * *   * ** * *  *      ** * * *    *  *  *** ** ******

P-NatCCd4.2     caacaggacgggcttgtttggcacagaaatgctatcgagattttagtttctttttcgacg     561
P-NatCCd4.1     tt---cccgacccccgtctggctatggaaacaaatcgggctttgagtttctagtccgacc     546
                            *  ** ****   * **    **** * *** *******  * ****

P-NatCCd4.2     cagcttagttga---atcggccgcatcagcttcatttggagttcgatttgatcctcaaga     618
P-NatCCd4.1     accaattatagtcagatgacattactccctgtaattggtagtcgggctccaaattcaga-     605
                     *  * *    **        **    * *** * ***  *  *  *   ***

P-NatCCd4.2     catactcactatgaagtattttggataaaatcta--aatacagccaaggttctaaaaaac     676
P-NatCCd4.1     -----gcccgatctgtttttatagaaaatatgttccccgactacccccgttttcaaaaaa     660
                      * * **    * ** * ** ** ** *      **  **   *** * *****

P-NatCCd4.2     gtaaggcatagaaaaacgtggaggtcgagcattgagcctgtac---ttgtttaagcgtac     733
P-NatCCd4.1     aatccattttttaaccttttgaaatattttataaaatgtttttttagtatttcggccagg     720
                        *   **    * **  *     **  *   * *      * ***  **

P-NatCCd4.2     taatgcataataaaagcctcatcaatgcgtactaaaacgcaagacacgatgaaacgtgaa     793
P-NatCCd4.1     agttgtcaagtt-tcgaccacccaatttgac-----------------cttttagctgcg     762
                   **   * *    * *    ****  *                    *   *  **

P-NatCCd4.2     gtccaagcttcaaacataagcgccccc--------taagtattg--cctagatgagattt     843
P-NatCCd4.1     cccctaacttataagaaaacaatttgttggttttagaactatttttattttatgtattta     822
                  ** * ***  ** * **                 **  ****     *  ***   **

P-NatCCd4.2     ttagaatactaaatacagtaacatgacaatattctggtttaacactgtctcatggaaaac     903
P-NatCCd4.1     tttattttcgaa--atattattttcaatatatcttcttataccatctaattatatctaga     880
                **    * * **  * * **   * *  ****  *  * ** **     * **    *

P-NatCCd4.2     cctgg---------ctgtcaaacgagggtacactagtgatg--ccc-acttgggatcaag     951
P-NatCCd4.1     catgatttagttaaatgtttcaagttcgtgacgtggtgacctggcaaagcttcaactaag     940
                * **           ***   * *   **    * ****     *  *  *   *  ***

P-NatCCd4.2     tacatagattatccacat--taggttatttgtccactaatatgctcttatttaacatctt     1009
P-NatCCd4.1     ccagtcgtttgtgtttttaaattttttcctcttaactaatttgctcttatttaaactct-     999
                    * * ** *     *      **   * *  ****** *************  ***

P-NatCCd4.2     ctgtctcccttcatttgtcagagtgtagataaatcctgcagaatttgtt-----------     1058
P-NatCCd4.1     taatctctcttcatttgtcacaaggtacatcaatctcctgaatcttgattatcaatttga     1059
                   **** ************ *  *** ** ****      *  *** *

P-NatCCd4.2     -gtcaagaaagatttgactatgggaacactt      1088
P-NatCCd4.1     cgcccaaaatgact--agcatgggaacactt      1088
                 * * * ** ** *  *  ************
```

Supplementary Fig. S3. Nucleotide sequence alignment of NatCCD4.1 and NatCCD4.2 promoters. The star codon is labelled in red. Conserved residues are depicted with an asterisk.

Fig. S4

```
NatCCD4.1       MVEENHETSILPEKAFSSRTTLQKLVQLIGKSTPR----KEV------VKKKKEPSLVAT  50
NatCCD4.2       MVSSVR-TEDKPQTTTTNGTAGREVAQPLKKETPPLPNPREILPSFLAPKKMESLLSPST  59
                **.. : *.  *:.: :. *: :::.* : *.**     :*:       ** :.    :*

NatCCD4.1       ILNAYEDFICTFLDLPLPPSLDPKHVLSGNYAAVDELPPTPCEVVEGALPSCLDGAYLRN  110
NatCCD4.2       IFNVVDNFINTCIDPPLHPSTDPKYVFLNNYAPVDELPPTQCQVVEGTLPTCLNGAYIRN  119
                *:*. ::** * :* ** ** ***:*: .*** ******* *:****:**:**:***:**

NatCCD4.1       GPNPQFIPRGPYHLFDGDGMLHVIKISQGKAVFCSRYVKTHKYIIEHKNGSPVIPSIFSS  170
NatCCD4.2       GPNPQFFPRGPYHTFEGDGMLHSIRIFNGKATFCSRYVKTYRYIVENKNGFPIFPNVFSG  179
                ******:****** *:****** *:* :***.********::**:*:*** *::*.:**.

NatCCD4.1       FNGFSATMARLVPFVVRSLAGQFNPSTHGIGVANTSLALIGGDLFALAESDLPYKLKLTP  230
NatCCD4.2       FNGLTALATRGALAAARILAGQFNPA-NGTGPANTSLALVGGKLYALGESDLPYTVKVAE  238
                ***::*  :* .  ..* *******: :* * *******:**.*:**.******.:*::

NatCCD4.1       DGDVITQGRHTFCSKPVMSMTAHPKTDPNTGEAFGFAYNVFHPFLTYFRINREGVKQKDI  290
NatCCD4.2       DGDIITQGRHDFDGKLIMSMTAHPKVDPDTKEAFAFRYGAISPFLTFFRINPDGTKQPDV  298
                ***:****** * .* :********.**:* ***.* *..: ****:**** :*.** *:

NatCCD4.1       PINSLTRSSFIHDFAVTKNYVVFPETQIVIDPMNIIRGKSPVGTDLAQVPRLGILPRYAE  350
NatCCD4.2       PIFSMKSPSLIHDFAITKKYAIFPEIQIGINPLKMMAGGSLIGTNPGKVPRLGVIPRYAK  358
                ** *:.  *:*****:**:*.:*** ** *:*:::: * * :**: .:*****::****:

NatCCD4.1       NDAEMTWIDAPGLNMVHAFNAWEEDDGNRVVILASNAFEFESPSDPMDFLHLSVDRVEIN  410
NatCCD4.2       DESEMKWFDVPGFNILHAINSWEEDDGDTIVLVAPNILSVEHTLERVDLIHLSVEKVKID  418
                :::**.*:*.**:*::**:*:******: :*::* * :..*   : :*::****::*:*:

NatCCD4.1       LKEKKVLRQPLCSTNLDLAVINPSYAGKKNRFIYAAVTTTTPKRAGVMKLDLSLFEADNR  470
NatCCD4.2       LKTGMVSRIPLSTRNLDFGAINPSYTGKKNKYVYAAIGDPMPKIPGVVKLDISVSGSDHR  478
                **   * * **.: ***:..*****:****:::***:    **  **:***:*:  :*:*

NatCCD4.1       DCAVGSRLYEPGCFGGEPFFVPRDPGSREDVDEDDGYLVTYLHNEKTGESRFLVMDAKSP  530
NatCCD4.2       DCIVASRLFGEGCLGGEPFFVAKEPDN-PSADEDDGYVVTYVHNEKNGESSFLVMDAKSP  537
                ** *.***:  **:******* ::*..  ..******:***:****.*** *********

NatCCD4.1       ELEIVAQVKLPGRVPYGFHGLFVPESELKDM  561
NatCCD4.2       NLDTVAAVKLPRRVPYGFHGIFVRENDLNKL  568
                :*: ** **** ********:** *.:*:.:
```

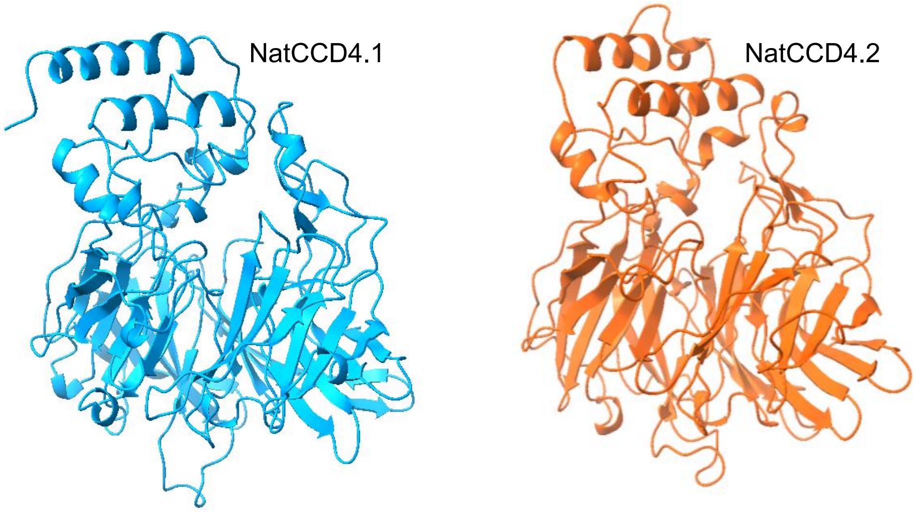

Fig. S4. Comparison of NatCCD4.1 and NatCCD4.2. A) Amino acid sequence alignment of NatCCD4.1 and NatCCD4.2. Conserved amino acid residues are depicted with asterisk, while two or one dost indicates conservation between groups of strongly or weakly similar properties, respectively. The amino acid residues involved in iron coordination are highlighted in yellow and blue, in yellow the conserved His residues. B)Tridimensional structures of NatCCD4.1 and NatCCD4.2. The 3D structure were predicted using Phyre2 software at intensive mode (http://www.sbg.bio.ic.ac.uk/phyre2/) and Chimera X (https://www.cgl.ucsf.edu/chimerax/).

Fig. S5

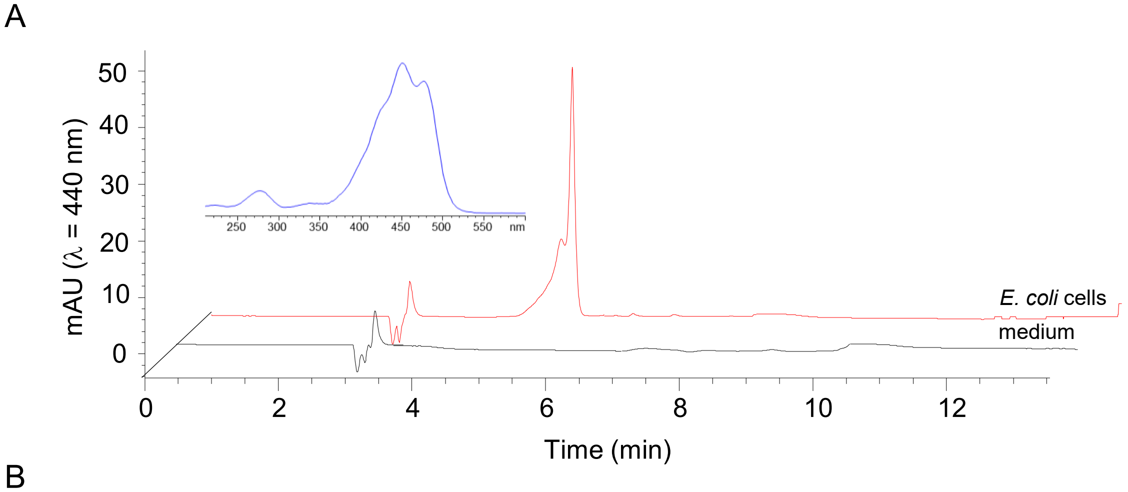

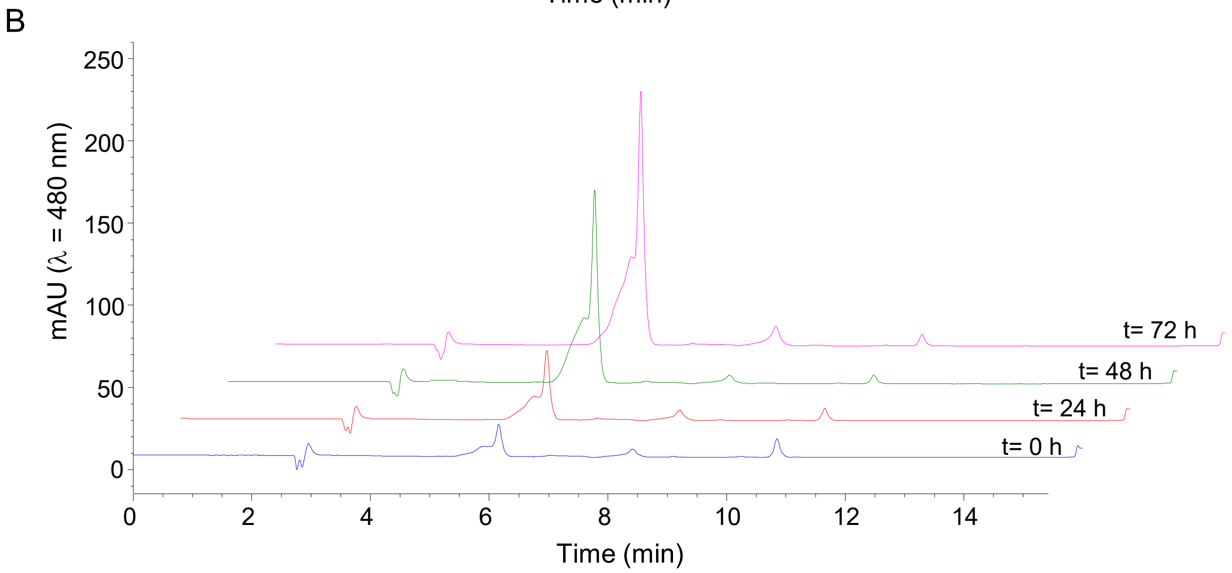

Supplementary Fig. S5. Lack of crocetin dialdehyde in cells pellet and medium in the two-phase experiment with n-dodecane. A) HPLC-DAD profile of apolar extracts from pellet of *E. coli* cells producing zeaxanthin and expressing NatCCD4.1 induced for 48 h with arabinose (0.2%, w/v) and with the addition of n-dodecane (16% v/v); and .HPLC-DAD profile of the medium in which these cells were grown. Inset is shown the spectra of zeaxanthin. B) HPLC-DAD profiles of apolar extracts from pellet of *E. coli* cells producing zeaxanthin and expressing NatCCD4.1 induced with arabinose (0.2%, w/v) and with the addition of n-dodecane (16% v/v) and collected at different times after induction.

Fig. S6

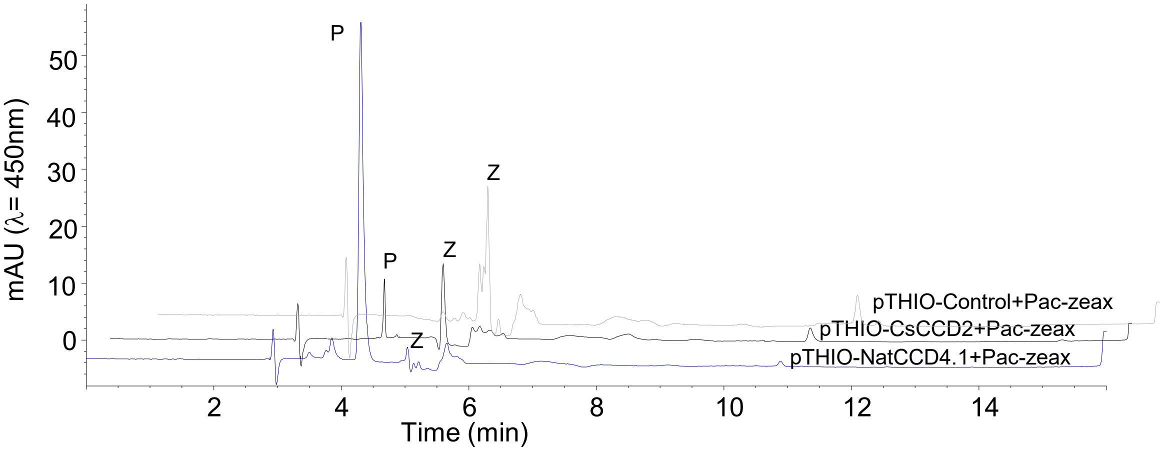

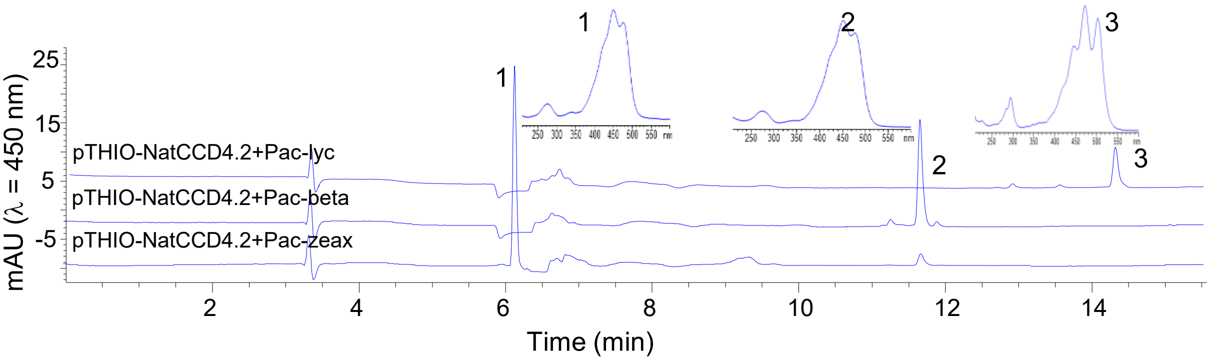

Supplemental Fig. S6. Activity assays of recombinant CCD enzymes in *E. coli* cells accumulating carotenoids. (A) Representative HPLC-DAD chromatograms of apolar extracts obtained from *E. coli* cells expressing NatCCD4.1, CsCCD2 and control cells. P= crocetin dialdehyde, Z= zeaxanthin. (B) Crocetin dialdehyde was not detected in the n-dodecane phase collected from bacterial cells acumulating different carotenoids and expressing NatCCD4.2. The induction of NatCCD4.2 was promoted by the addition of arabinose (0.2% w/v), and the n-dodecane pase collected 24 h after induction. The experimentes were repeated independently three times with the same negative results. Number correspond to the substrates used in the assay. 1: zeaxanthin, 2: β-carotene, and 3: lycopene.

Fig. S7

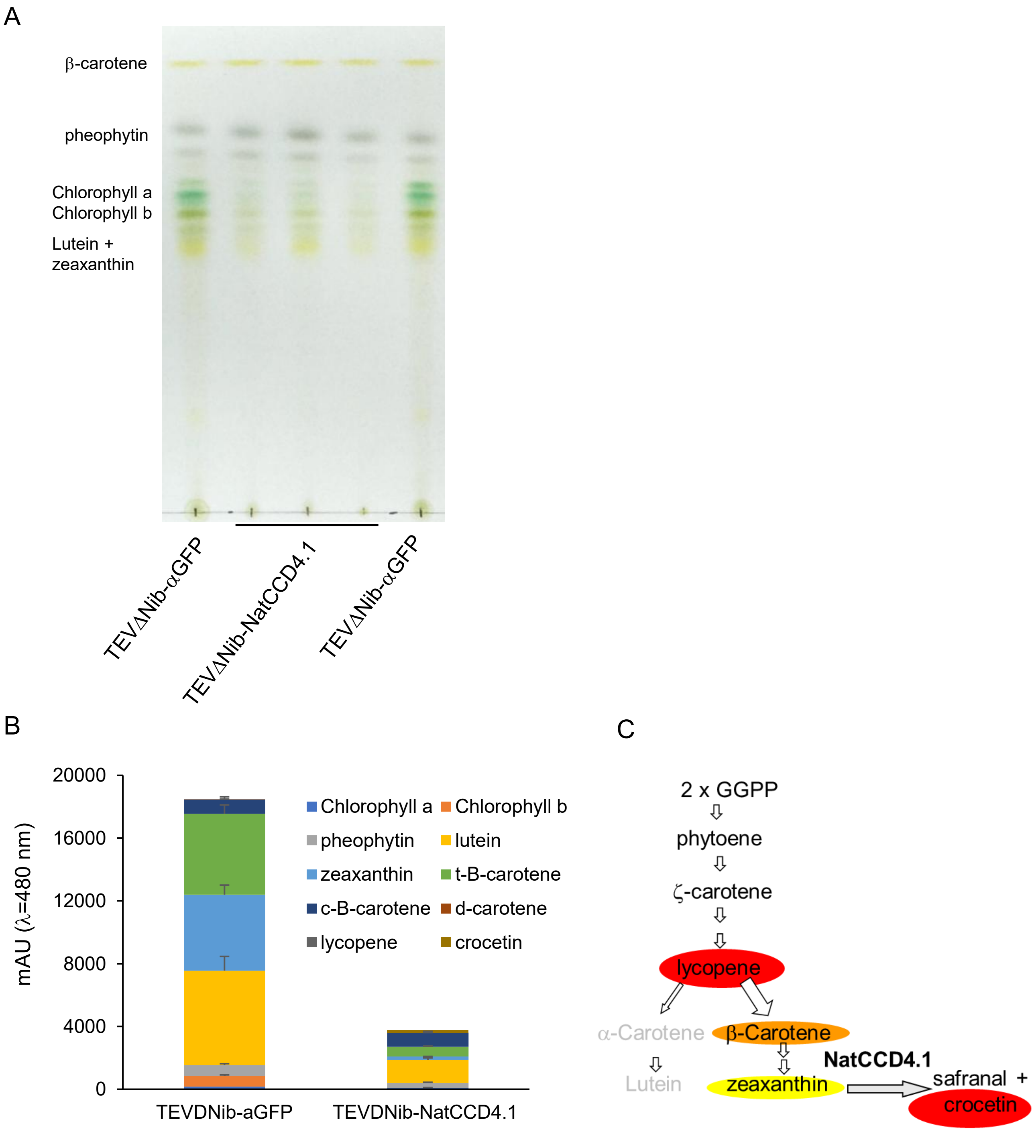

Supplemental Fig. S7. Analyses of apolar extracts from *N. benthamina* experiments. A) TLC analyses of pigments extracted from *N. benthamina* leaves with 2:1 methanol:chloroform. B) Levesl of carotenoids, chlorophylls and crocetin in the apolar extracts obtained from *N. benthamina* leaves . Data are averages ± SD of three biological replicates. Analyses were performed at 14 dpi. C) Schematic representation of the carotenoid pathway showing how NatCCD4.1 activity reduced notably the levels of the substrate used, zeaxanthin, and the levesl of the zeaxanthin precursor : β-carotene.

Fig. S8

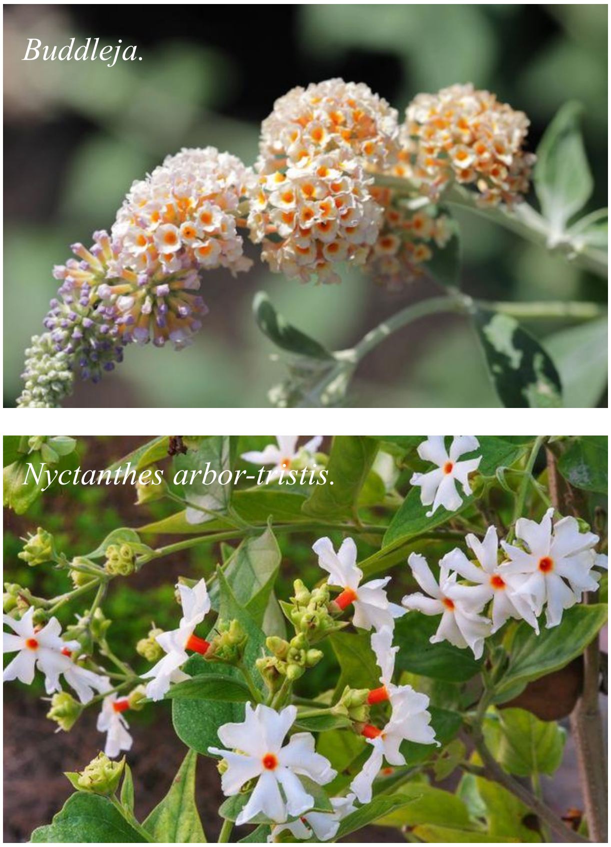

Supplemental Fig. S8. Crocins accumulated in the calisxof the flowers of *Buddleja* and *Nyctanthes arbor-tristis.*
