# Supplementary material for: Biotechnological production of crocetin and crocins using a carotenoid cleavage dioxygenase (CCD4) from Nyctanthes arbor-tristis
Source: Front Plant Sci. 2025 Oct 17;16:1671592. doi: 10.3389/fpls.2025.1671592 (PMC12575119; doi:10.3389/fpls.2025.1671592)
Supplement: Supplementary file 2 [file SupplementaryFile1.pdf]

Table S1. Oligonucleotides used for cloning and activity assays of *NatCCD4.1* and *NatCCD4.2*.

gene name	Forward	Reverse
	Oligonucleotides for amplification from synthesized gene	
NatCCD4.1	ATGACTAGCATGGGAACACTTTCTTCT	TTACGTCTACATGTCTTTAAGTTCACTTTCTGG
NatCCD4.2	ATGGGAACACTTTCTTCTTCTTTC	CTAATTATGAAAATTCCTAAGGT
	Oligonucleotides for activity assays in E. coli	
NatCCD4.1	CGCCCTTGGCGAATTCATGACTAGCATGGGAACACTTTCTTCT	TACCCTCGAGGAATTCTTACGTCTACATGTCTTTAAGTTCACTTTCTGG
NatCCD4.2	CGCCCTTGGCGAATTCATGGGAACACTTTCTTCTTCTTTCCT	TACCCTCGAGGAATTCCTAATTATGAAAATTCCTAAGGTTTTTAAGTTCACTTTCTGG
	Oligonucleotides for activity assays in *N. benthamiana*	
NatCCD4.1	ACCATTTACGAACGATAGCCATGAACACATTTTCTTCCTCTTTCC	AAATATAAATTTTCGGTTGTCAGCTTGTCGAGCTCGGTC
